# Multifaceted Functions and Novel Insight Into the Regulatory Role of RNA N^6^-Methyladenosine Modification in Musculoskeletal Disorders

**DOI:** 10.3389/fcell.2020.00870

**Published:** 2020-09-02

**Authors:** Wenchao Zhang, Lile He, Zhongyue Liu, Xiaolei Ren, Lin Qi, Lu Wan, Wanchun Wang, Chao Tu, Zhihong Li

**Affiliations:** ^1^Department of Orthopedics, The Second Xiangya Hospital, Central South University, Changsha, China; ^2^Hunan Key Laboratory of Tumor Models and Individualized Medicine, The Second Xiangya Hospital, Central South University, Changsha, China; ^3^Department of Cardiovascular Surgery, The Second Xiangya Hospital, Central South University, Changsha, China

**Keywords:** RNA N^6^-methyladenosine, METTL3, FTO, musculoskeletal disorders, epigenetics

## Abstract

RNA modifications have emerged as key regulators of transcript expression in diverse physiological and pathological processes. As one of the most prevalent types of RNA modifications, N^6^-methyladenosine (m^6^A) has become the highlight in modulation of various diseases through interfering RNA splicing, translation, nuclear export, and decay. In many cases, the detailed functions of m^6^A in cellular processes and diseases remain unclear. Notably, recent studies have determined the relationship between m^6^A modification and musculoskeletal disorders containing osteosarcoma, osteoarthritis, rheumatoid arthritis, osteoporosis, etc. Herein, this review comprehensively summarizes the recent advances of m^6^A modification in pathogenesis and progression of musculoskeletal diseases. Specifically, the underlying molecular mechanisms, detection technologies, regulatory functions, clinical implications, and future perspectives of m^6^A in musculoskeletal disorders are discussed, with the aim to provide a novel insight into their association.

## Introduction

Currently, a growing number of studies have shed light on a new hereditary manner, the epigenetics, which refers to changes in phenotype without DNA or RNA sequences alteration (Harvey et al., 2018). Several epigenetics manners have been identified, comprising the histone modification, DNA and RNA methylation, and noncoding RNA (ncRNA) modification. Of note, there have been more than 100 modifications demonstrated within RNAs over the past few years, including the N^1^-methyladenosine (m^1^A), m^6^A, 5-methylcytosine (m^5^C), 7-methylguanosine (m^7^G), m^1^G, m^2^G, m^6^G, etc. Among them, the m^6^A has received considerable attention because of its high abundance. It relates to a dynamic and reversible RNA modification that participate in a wide range of biological and pathological processes, such as the cancer progression (Lan et al., 2019) and inflammation (Zong et al., 2019). Specifically, it can manipulate the RNA splicing, export, translation, and degradation through methylation and demethylation mediated by multiple enzymes (Cao et al., 2016).

Musculoskeletal disorders are a set of prevalent diseases characterized by dysfunction of bone and skeletal muscle, including, but not limited to, osteosarcoma (OS), osteoarthritis (OA), rheumatoid arthritis (RA), osteoporosis (OP), etc. (Madan and Grime, 2015). Several epigenetics manners have been investigated in this field (Tu et al., 2019; van Wijnen and Westendorf, 2019). Among them, the alteration of m^6^A modification has been associated with the initiation and progression of musculoskeletal diseases.

In this review, we broadly summarize the functional repertoire of m^6^A in various musculoskeletal disorders, aiming to expand our understanding and discuss the putative perspective for adopting m^6^A as a novel biomarker and therapeutic target in musculoskeletal diseases.

## RNA M^6^A Modification

**FIGURE 1:**
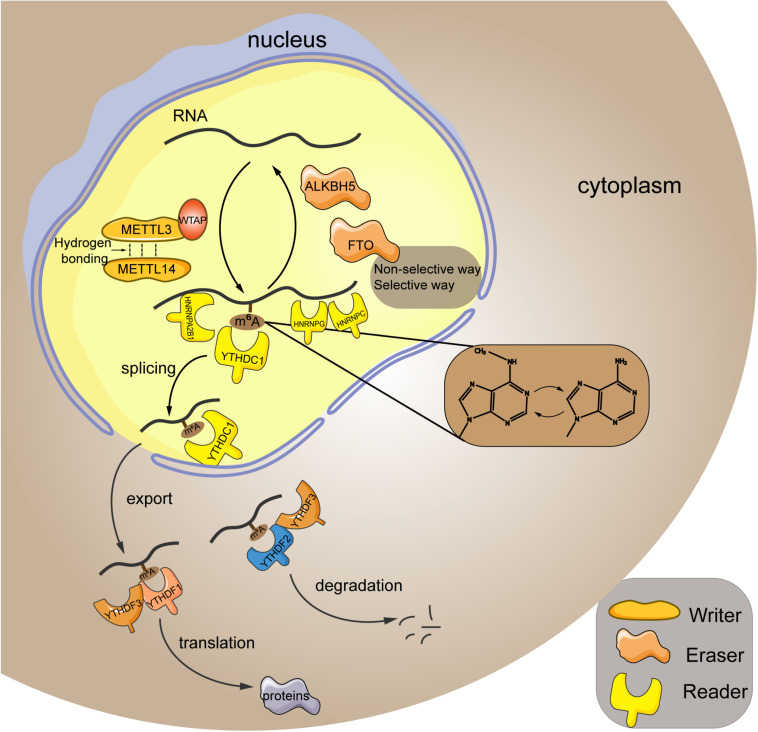
Mechanism of m^6^A RNA modification. M^6^A modification is a reversible process mediated by its regulatory proteins, including the writers (MELTT3, METTL14, WTAP, etc.), erasers (FTO, ALKBH5, etc.), and readers (YTHDF1, YTHDF2, YTHDF3, etc.). METTL3, methyltransferase-like 3; METTL14, methyltransferase-like 14; WTAP, Wilms tumor 1-associated protein; FTO, fat mass and obesity-associated protein; ALKBH5, alkB homolog 5; YTHDF1, YTH domain family; member 1; HNRNPA2B1, heterogeneous nuclear ribonucleoprotein A2B1; HNRNPC, heterogeneous nuclear ribonucleoprotein C; HNRNPG, heterogeneous nuclear ribonucleoprotein G.

As aforementioned, m^6^A modification is a dynamic and reversible epigenetic change (Zhang et al., 2020), which affects the stability and function of RNAs, thereby modulating the pathogenesis and progression of diseases (Qin et al., 2020; Wang et al., 2020; Zhu et al., 2020). M^6^A modification has been identified in more than 7,000 human genes, and it preferentially occurred at the site of stop codons and long internal exons within the RRACH sequence (R = G or A; H = A, C, or U) (Dominissini et al., 2012). Based on current evidences, m^6^A modification is able to interfere in RNA processing, splicing, export, degradation, and translation through the “writers,” “erasers,” and “readers” proteins (Chen et al., 2019). The detailed graphical description of the RNA m^6^A mechanism is presented in Figure 1.

Writers refer to the RNA methyltransferases, including methyltransferase-like 3 (METTL3), methyltransferase-like 14 (METTL14), methyltransferase-like 16 (METTL16), Wilms tumor 1-associated protein (WTAP), RNA-binding motif 15, etc. They are in charge of installing m^6^A to the RNA strand. In particular, METTL3 and METTL14 are the most studied key regulators in this process. They contain an S-adenosylmethionine–binding motif and are capable of adding methyl to the adenosine. Meanwhile, these two proteins can form a heterodimer core complex to modulate the cellular m^6^A deposition (Liu et al., 2014), in which they adopted a class I methyltransferase fold and interacted with each other through hydrogen bonding. Additionally, biochemical analysis revealed that METTL3 principally acted as the catalytic core, whereas METTL14 mainly functioned as an RNA-binding platform (Wang et al., 2016). Besides, the cofactor WTAP itself shows no methyltransferase activity, whereas it could interplay with the core complex to mediate its localization in nuclear spots (Ping et al., 2014). Furthermore, it has been reported that WTAP protein homeostasis in turn relied on the METTL3 protein levels (Sorci et al., 2018). Therefore, these writers cooperate to exert their function of m^6^A methylation within the specific RNA sites.

In contrast to the “writers,” the “erasers” are demethylases that function in methyl removal, canonically involving the fat mass and obesity-associated protein (FTO) and alkB homolog 5 (ALKBH5). FTO can oxidize m^6^A to generate the N(6)-hydroxymethyladenosine and N(6)-formyladenosine that have a half-life time less than 3 h (Fu et al., 2013). Generally, FTO erase methyl in either the selective or nonselective way. In the selective way, FTO recognizes and binds to the specific m^6^A-containing motif in cells (Li et al., 2019a). Because the association between FTO and RNA is weak, the additional cellular factors can corporately work with FTO to recognize and choose the target sites (Li et al., 2019a). In addition, the m^6^A itself can act as a “conformational marker” and interfere with the interaction between m^6^A and FTO via altering conformational outcomes in RNAs, which is the nonselective way of FTO-mediated demethylation (Zou et al., 2016). ALKBH5 is a 2-oxoglutarate and ferrous iron–dependent nucleic acid oxygenase that induces the demethylation of multiple RNAs. It was illuminated that ALKBH5 could occupy a similar region as L1 loop of the FTO protein that was associated with the single-strand RNA selectivity (Aik et al., 2014). Taken together, FTO and ALKBH5 are both the core regulators of m^6^A demethylation.

Readers are a group of proteins that discern the m^6^A modification and determine the functions of RNA transcripts. YTH domain (YTHD) family members (consisting of YTHDF1, YTHDF2, YTHDF3, YTHDC1, and YTHDC2) constitute a large class of m^6^A readers. They are located in nucleus or cytoplasm (specifically, YTHDF1 and YTHDF2 are located both in cytoplasm and nucleus, whereas YTHDF3 is only found in the cytoplasm) (Reichel et al., 2019), and characterized by containing the YT521B homology (YTH) domain that possesses an exquisite pocket with two conserved tryptophan residues (W377 and W428) for specific recognition of the methyl group (Xu et al., 2014). YTHDF1 is shown to promote translation of mRNA and enhance protein synthesis through impacting on the translation machinery, ensuring the sufficient protein generation is marked by m^6^A (Wang et al., 2015). Conversely, YTHDF2 mediates the degradation of its target m^6^A transcripts via reducing their stability (Li et al., 2018). Additionally, YTHDF3 participates in either promoting the protein synthesis in synergy with YTHDF1 or facilitating RNA degradation via interaction with YTHDF2. Thus, these three YTHDF proteins read the m^6^A modification in a cooperative way (Ni et al., 2019). Moreover, the nuclear YTHDC1 is associated with the RNA splicing (Kasowitz et al., 2018) and the export of m^6^A modified RNAs from nucleus to cytoplasm (Roundtree et al., 2017), whereas YTHDC2 engages in the elongation-promoting effect of m^6^A methylated RNA coding region (Mao et al., 2019). In addition to the YTHD family, the family of heterogeneous nuclear ribonucleoproteins (HNRNPs) is another set of m^6^A readers, which binds to pre-mRNA to interfere its stability and splicing (Geuens et al., 2016). HNRNPA2B1 has been well-accepted as a nuclear reader of m^6^A, binding to the RGm^6^AC containing sites on RNA to affect the alternative splicing and the processing of miRNAs (Alarcón et al., 2015). As to HNRNPC, because the m^6^A residues within RNA strand can destabilize the RNA duplexes (Kierzek and Kierzek, 2003), the structure of RNA may be altered when it is m^6^A modified. Given this, the m^6^A has been proposed to make the UUUUU tract within RNA to become more unfolded and accessible to HNRNPC (Liu et al., 2015), which is termed as the “m^6^A switch.” Moreover, HNRNPG also recognizes the m^6^A through “m^6^A switch.” HNRNPG has a low-complexity region that can discern a specific motif exposed by m^6^A-mediated RNA structural change (Liu et al., 2017), subsequently modulating the cotranscriptional pre-mRNA splicing (Zhou et al., 2019). Furthermore, the insulin-like growth factor 2 mRNA-binding proteins (IGF2BPs) can target the mRNA transcripts in an m^6^A-dependent way through recognizing their GG(m^6^A)C sequences, subsequently stabilizing the targeted RNAs under both the normal and stress conditions (Huang et al., 2018).

## Advances in Technologies for M^6^A Detection

Currently, a massive number of technologies have been developed for m^6^A detection based on the immunohistochemistry or hybridization properties (Ovcharenko and Rentmeister, 2018). According to their detection performance, these technologies can be classified into the semiquantitative, quantitative, and precise location detection methods (Zhu et al., 2019; Table 1).

**TABLE 1:** Technologies for m^6^A detection.

**Classification**	**Detection method**	**Mechanism**	**Advantages**	**Disadvantages**	**References**
Semiquantitative	Dot blot	Using antibodies that specifically bind to the m^6^A	Simplicity, speediness	Not quantitative, sensitivity is low when the m^6^A RNA fragment is small	Nagarajan et al., 2019; Zhu et al., 2019
	Methyl sensitivity of MazF RNA endonucleases	MazF selectively cleave the 5′-ACA-3′ but not the 5′-(m^6^A)CA-3′ sequence	Simple analyses of both m^6^A demethylase and methyltransferase activity	Not quantitative, only able to cleave the 5′-ACA-3′ site in single-stranded RNA	Imanishi et al., 2017
	Immuno-Northern blot	Immunoblotting using antibodies against modified nucleosides	High specificity, sensitivity, and potential quantitative capability	Not quantitative	Mishima et al., 2015
Quantitative	Photo-crosslinks based quantitative proteomics	Stabilizing protein–RNA interaction and detecting via synthetic probes	Quantitative, high efficiency	Requirement of the synthesis of the probe	Arguello et al., 2017
	Electrochemical immunosensor method	Antibody–antigen interaction	Simplicity, low-cost, high specificity and sensitivity	N/A	Yin et al., 2017
	Support vector machine–based method	Computational prediction based on existent high-throughout data	Simplicity, saving the experiment cost	Relying on the existent experimental data	Chen W. et al., 2016
Gene specific location detection	MeRIP-Seq	Combination of the ChIP-Seq and RNA-Seq	High-throughput	Poor reproducibility	Meyer et al., 2012; McIntyre et al., 2020
	m^6^A-LAIC-seq	Sequencing intact full-length RNA in both m^6^A-positive and -negative fractions post-RIP	Detecting m^6^A in differential RNA isoform, determining the m^6^A levels in each gene	Cannot stoichiometrically analyze the methylation of a single modified nucleotide	Molinie et al., 2016
Nucleotide-specific location detection	HRM	Detecting the alteration of nucleic acid duplex melting properties	High-throughput, high-resolution	Relying on the previous knowledge of the modified nucleoside position at a particular RNA site	Golovina et al., 2014
	SCARLET	RNAs are cleaved, radiolabeled, digested, and analyzed by TLC	High-resolution	N/A	Liu et al., 2013
	miCLIP	Inducing specific mutational signatures to m^6^A antibody	High-resolution	N/A	Linder et al., 2015

*HRM, high-resolution melting; m^6^A-LAIC-seq, m^6^A level and isoform-characterization sequencing; MeRIP-Seq, methylated RNA immunoprecipitation next generation sequencing; miCLIP, m^6^A individual nucleotide resolution cross linking and immunoprecipitation; N/A, not available; SCARLET, site-specific cleavage and radioactive labeling followed by ligation-assisted extraction and thin-layer chromatography.*

### Semiquantitative Methods

Semiquantitative detection strategies, including the dot blot, methyl sensitivity of MazF RNA endonucleases, and immune-Northern blot, are used to determine the presence of m^6^A modification rather than the amount. Among them, dot blot is applied to detect the global change of m^6^A by using antibodies that specifically bind to the m^6^A site. It is relatively simple and fast but not quantitative (Zhu et al., 2019), with low sensitivity when the m^6^A RNA fragment is small in the samples (Nagarajan et al., 2019). A modified dot blot method has been adopted to increase the sensitivity through adding an immunoprecipitation step to enrich the m^6^A RNA before detection (Nagarajan et al., 2019). Further, *Escherichia coli* MazF is a sequence-specific endoribonuclease, which cleave the 5′-ACA-3′ sequence but not the 5′-(m^6^A)CA-3′ sequence within RNA strand, whereby it is an m^6^A-sensitive RNA cleavage enzyme. Based on this technology, a new high-throughput detection method for m^6^A has been established by researchers (Imanishi et al., 2017). Immuno-Northern blot is another way for semiquantitative detection of various types of RNA modifications. In this way, RNAs are separated and transferred onto a nylon membrane, followed by immunoblotting for measurement (Mishima et al., 2015).

### Quantitative Methods

Unlike semiquantitative methods, the quantitative methods, including photo-crosslinks–based quantitative proteomics, electrochemical immunosensor method, and support vector machine–based method, can be utilized to determine the amount of m^6^A RNA. Arguello et al. (2017) have proposed that the photo-crosslinkers, a widely used method to stabilize the protein–RNA interaction, could combine with quantitative proteomics to detect m^6^A RNA. Meanwhile, a diazirine containing RNA probes has been recently synthesized to improve its efficiency (Arguello et al., 2017). Moreover, in the electrochemical immunosensor method (Yin et al., 2017), an anti-m^6^A antibody has been applied to recognize and capture the m^6^A-5′-triphosphate. Silver nanoparticles and amine-PEG3-biotin functionalized SiO_2_ nanospheres (Ag@SiO_2_) were used to amplify the signal, and phos-tag-biotin was employed as a bridge to connect the m^6^ATP and Ag@SiO_2_. This approach is convenient, low-cost, and of high specificity and sensitivity. Besides, the support vector machine–based method is a computational way to predict the m^6^A site within RNA strand based on the existent high-throughput experiment data (Chen W. et al., 2016).

### M^6^A Location Detection Methods

To figure out the specific location of m^6^A within RNAs, several detection methods have been further developed by researchers (Ovcharenko and Rentmeister, 2018). Generally, these techniques can be divided into two sorts, namely, the gene-specific and the nucleoside-specific detection method. The former one comprises methylated RNA immunoprecipitation next-generation sequencing (MeRIP-Seq), m^6^A level and isoform-characterization sequencing (m^6^A-LAIC-seq), etc. While the latter contains high-resolution melting (HRM) analysis, site-specific cleavage, and radioactive labeling followed by ligation-assisted extraction and thin-layer chromatography (SCARLET), m^6^A individual nucleotide resolution cross-linking and immunoprecipitation (miCLIP), etc.

MeRIP-Seq combines the ChIP-Seq and RNA-Seq (Meyer et al., 2012), in which the anti-m^6^A antibody is incubated with the RNA fragments, and the precipitated fragments then are sequenced. Hence, it can determine the origin of m^6^A at a gene level. However, a more recent study reported that this method was of poor reproducibility because it was easily influenced by the noise (McIntyre et al., 2020). On the basis of MeRIP-seq, m^6^A-LAIC-seq (Molinie et al., 2016) is further developed to detect the dynamic range and isoform complexity of m^6^A content in a single gene, in which the intact full-length RNAs in both m^6^A-positive and m^6^A-negative fractions post-RIP are sequenced, and thus the differential isoform usages in each transcripts are detectable.

HRM (Golovina et al., 2014) is a high-throughput method for m^6^A detection at a specific site within the RNA strand. This method works by detecting the alteration of nucleic acid duplex melting properties caused by m^6^A modification. For instance, it has been observed that the melting temperature of RNA–DNA duplex was reduced by the presence of m^6^A modification (Golovina et al., 2014). SCARLET (Liu et al., 2013) is a method that enables detection of m^6^A status at any site in mRNA/long ncRNA (lncRNA), in which the m^6^A-containing candidate sites are cleaved, radiolabeled, and site-specific ligated, followed by complete nuclease digestion. The digested m^6^A residue is then measured by thin-layer chromatography (TLC). Moreover, MiCLIP can be used to determine the m^6^A site at a nucleotide-specific level (Linder et al., 2015). M^6^A antibodies bind to the m^6^A sites within RNA strands, and the m^6^A residues are then located by inducing specific mutational signatures after ultraviolet light–induced antibody-RNA cross-linking and reverse transcription.

## RNA M^6^A Methylation in Musculoskeletal Biology

**FIGURE 2:**
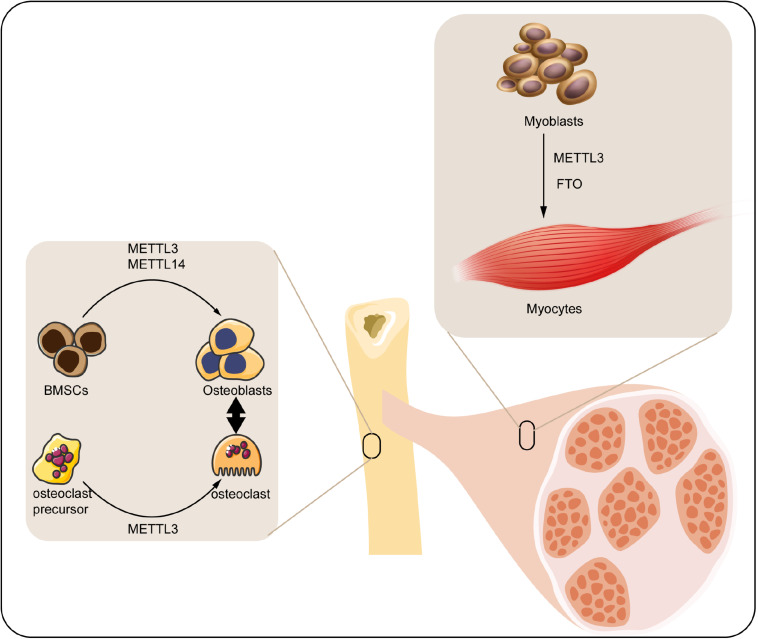
Roles of m^6^A modification in musculoskeletal biology. M^6^A regulatory proteins participate in differentiation of BMSCs, osteoclast precursors, and myoblasts. METTL3, methyltransferase-like 3; METTL14, methyltransferase-like 14; FTO, fat mass and obesity-associated protein; BMSCs, bone marrow mesenchymal stem cells.

Musculoskeletal system mainly consists of skeleton and skeletal muscle, which directly participates in the motor function of human body. Diverse transcriptional factors have been reported to involve in the genesis and maintenance of musculoskeletal system. M^6^A modification is a widely discovered and annotated epigenetic manner that takes part in the biology of musculoskeletal system. Herein, we discuss the mechanism and regulatory function of m^6^A in this process (Figure 2).

### M^6^A in Bone Remodeling

Bone is a connective tissue functioning in mechanical support, mineral homeostasis, hematopoiesis, etc., which maintains its metabolic balance principally through bone remodeling, a dynamic process involved in the formation of bone matrix through osteoblasts and removal of bone mass via osteoclasts (Hadjidakis and Androulakis, 2006). Osteoblast is derived from the bone marrow mesenchymal stem cells (BMSCs) under the impact of numerous regulators, which can produce matrix to form the bone tissue. With the development of bone matrix, some osteoblasts finally reorganized and embedded into the matrix as osteocytes (Blair et al., 2017). Accordingly, osteocytes play a vital role in monitoring the bone quality and sensing the mechanotransduction, as well as secreting regulatory factors associated with bone anabolism (Tresguerres et al., 2020). By contrast, osteoclasts are multinuclear cells derived from myeloid precursors, which function in degradation and resorption of bone through secreting proteolytic enzymes and acid (Charles and Aliprantis, 2014). The osteoblasts and osteoclasts couple with each other to maintain the dynamic homeostasis of bones (Weivoda et al., 2016). Any impairment of the homeostasis under pathological condition may contribute to the bone disorder.

As an indispensable part of epigenetic regulation, m^6^A modification is suggested as a crucial regulator participating in either the osteogenic or osteoclastogenic processes of bone. For instance, the expression of m^6^A methyltransferases (METTL3 and METTL14) was found significantly elevated in BMSCs undergoing osteogenic induction. Accordingly, knockdown of METTL3 reduced the mRNA level of genes related to BMSC proliferation and differentiation such as the Vegfa-164 and Vegfa-188 (Tian et al., 2019). Apart from this, MYD88 gene, a vital upstream regulator of nuclear factor κB (NF-κB) signaling, was methylated by METTL3, followed by activation of NF-κB and repression of osteogenic progression. Meanwhile, the METTL3-mediated osteogenic differentiation tendency could be reversed by demethylase ALKBH5 (Yu et al., 2020). Moreover, silencing METTL3 decreased the osteogenic markers, Smad signaling, and mineralized nodules in preosteoblast MC3T3-E1 cells, indicating the reduction of osteoblast differentiation (Zhang Y. et al., 2019). Hence, the METTL3-mediated m^6^A methylation significantly contributes to the maintenance of osteogenesis. Conversely, METTL3-mediated m^6^A methylation was also reported to facilitate the osteoclast differentiation. METTL3 level was elevated during osteoclastogenesis, and METTL3 depletion suppressed the differentiation and bone-resorbing ability of osteoclasts. Mechanistically, Atp6v0d2 mRNA, the principal regulator of osteoclast precursor cells fusion (Kim et al., 2009) was stabilized by the m^6^A-binding protein YTHDF2 when the METTL3 was abolished (Li et al., 2020).

Taken together, the present studies have implicated the function of METTL3-mediated m^6^A in either the osteogenic or the osteoclastogenic differentiation of bone remodeling.

### M^6^A in Skeletal Muscle Regulation

Skeletal muscle comprises almost 40% of the total body weight, which functions in both the mechanical and metabolic processes of the body such as force generation and heat production (Frontera and Ochala, 2015). Retaining skeletal muscle mass is crucial for its physiological function, which is principally determined by the size and amount of muscle fibers. Skeletal muscle fibers are generated from myoblasts via myoblasts fusion, a process named as myogenesis (Sampath et al., 2018). Epigenetic modifications involving the histone modification (Machado et al., 2017), ncRNA (Liu M. et al., 2019), DNA methylation (Miyata et al., 2015), etc. have been unraveled to play a part in this process. Among them, m^6^A was proposed as a concernful regulator.

In the previous analyses, m^6^A modification can modulate the activity of skeletal muscle via modifying the muscle mass and interfering with the myoblasts differentiation: (1) modifying the muscle mass. For instance, upregulation of FTO gene expression was closely associated with skeletal muscle mass increase in overweight individuals (Doaei et al., 2019). Moreover, maternal high-fat intake could even disturb the m^6^A modification and FTO gene expression in skeletal muscle of its offspring (Li et al., 2016). Wu et al. (2017) have confirmed that activation of AMPK decreases the lipid accumulation in skeletal muscle cells through inversely regulating FTO expression and FTO-mediated demethylation. (2) Interfering with the myoblasts differentiation. It has been elucidated that FTO downregulation inhibited the myoblasts differentiation of mice through affecting the activity of mTOR-PGC-1α-mitochondria axis, which suppressed the mitochondria biogenesis and energy production of skeletal muscle cells (Wang et al., 2017). In addition, myoblast differentiation was regulated by METTL3-promoted MyoD. Specifically, suppression of METTL3 downregulated the activation of MyoD by modifying the sites within 5’ untranslated region of MyoD mRNA (Kudou et al., 2017).

## Roles of M^6^A in Musculoskeletal Disorders

**FIGURE 3:**
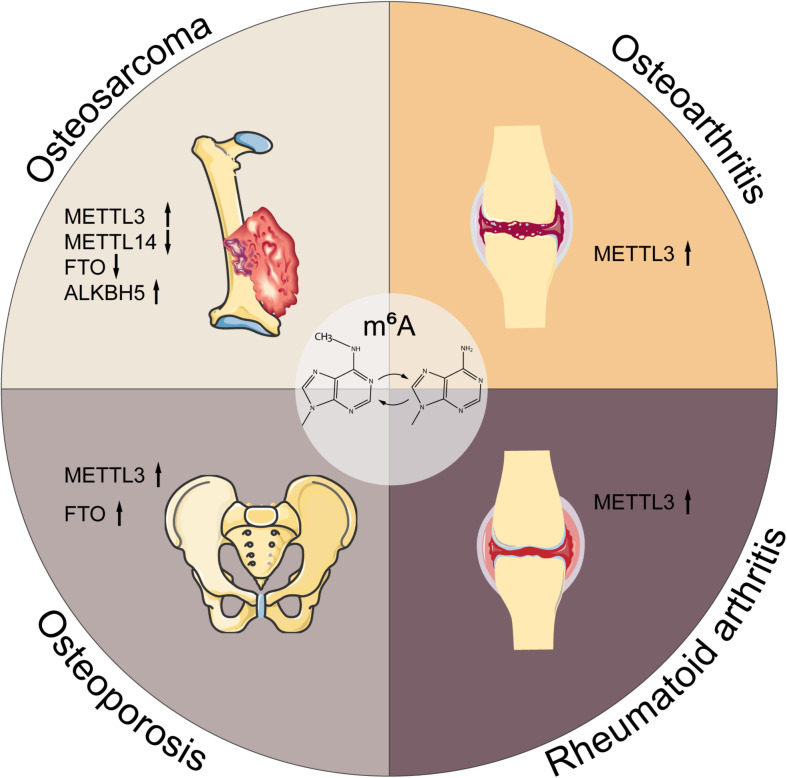
M^6^A in musculoskeletal disorders. M^6^A is associated with the progression of multiple musculoskeletal diseases, including osteosarcoma, osteoarthritis, rheumatoid arthritis, and osteoporosis. METTL3, methyltransferase-like 3; METTL14, methyltransferase-like 14; FTO, fat mass and obesity-associated protein; ALKBH5, alkB homolog 5; YTHDF1, YTH domain family, member 1.

Musculoskeletal disorders refer to abnormalities of skeleton or skeletal muscle, emerging as the tumor, inflammation, trauma, etc., which may lead to disability and paralysis. Although previous work has shed light on the roles of m^6^A in musculoskeletal disorders, the details of m^6^A function and its involvement in the pathogenesis and progression remain unclear. Here, we summarize the current evidences concerning the pleiotropic functions of m^6^A in musculoskeletal diseases, as presented in Figure 3 and Table 2.

**TABLE 2:** The role of m^6^A in musculoskeletal disorders.

**Diseases**	**M^6^A component**	**Samples and condition**	**Function**	**Regulation**	**Target**	**Role in disease**	**References**
OS	METTL14, METTL3, FTO, ALKBH5	MG63/DXR (doxorubicin treatment)	Oncogene	↓:METTL14/FTO; ↑:METTL3/ALKBH5	Wnt signaling	Emergence and maintaining of OSCs, promoting drug resistance	Wang Y. et al., 2019
	ALKBH5	70 pairs of OS and normal tissues Osteoblast cell line and OS cell lines	Oncogene	↑	PVT1	Promoting OS cell proliferation	Chen et al., 2020
	METTL3	HOS, SAOS-2, U2OS, and MG63 cells	Oncogene	↑	ATAD2	Enhancing OS cell growth and metastasis	Zhou et al., 2020
	METTL3	28 pairs of OS cancerous and paracancerous tissues; hFOB1.19 and OS cell lines	Oncogene	↑	DRG1	Promoting OS progression	Ling et al., 2020
	METTL3	40 paired of OS tissues and adjacent tissues Osteoblast cells and OS cell lines	Oncogene	↑	LEF1	Promoting OS progression	Miao et al., 2019
OA	METTL3	ATDC5 cells	promotor	↑	NF-κB signaling	Promoting inflammatory response, collagen synthesis and degradation, and cell apoptosis in chondrocytes	Liu Q. et al., 2019
RA	METTL3	47 RA patients and 30 health controls; THP-1 cell	promotor	↑	NF-κB signaling	Promoting RA disease activity	Wang J. et al., 2019
OP	METTL3	METTL3 knock-out and knock-in mice; primary MSCs	Suppressor	↓	PTH/Pth1r signaling	Impairing bone formation	Wu et al., 2018
	METTL3	Female OP patients and control subjects; METTL3^+/–^ mice	Suppressor	↓	miR320/RUNX2	Promoting OP development	Yan et al., 2020
	FTO	44 female/male individuals with/without OP Young and aged C57BL/6J mice	promotor	↑	Pparg	Promoting the shift of osteoporotic BMSC fate to adipocyte	Shen et al., 2018
	FTO	FTO^flx/flx^ (FTO^f/f^) mice	Suppressor	↑	ER stress pathway	Protecting osteoblasts from genotoxic damage Maintaining bone mass	Zhang Q. et al., 2019

*↑, Upregulation; ↓, Downregulation; ALKBH5, alkB homolog 5, ATAD2, ATPase family AAA domain containing 2; DRG1, GTP-binding protein 1; ER, endoplasmic reticulum; FTO, fat mass and obesity-associated protein; LEF1, lymphoid enhancer binding factor 1; METTL3, methyltransferase-like 3; METTL14, methyltransferase-like 14; NF-κB, nuclear factor κB; OA, osteoarthritis; OP, osteoporosis; OS, osteosarcoma; Pparg, peroxisome proliferator-activated receptor γ; PTH/Pth1r, parathyroid hormone/parathyroid hormone receptor-1; PVT1, plasmacytoma variant translocation 1; RA, rheumatoid arthritis; RUNX2, runt-related transcription factor 2.*

### M^6^A in OS

OS is the most common primary malignant bone tumor that mainly occurs in teenagers and adolescents with an annual incidence of 3.1 case per million (Gianferante et al., 2017). Although considerable advancement has been achieved in the past decades, the comprehensive mechanism network of OS has not yet been fully investigated. Currently, the standardized therapy for OS is limb salvage surgery or amputation combined with multiregimen-based chemotherapy. However, the survival rate of patients is unsatisfactory because of potential chemoresistance, lung metastasis, or tumor relapses. Recently, increasing studies have explored new therapeutic strategies for OS such as the molecular target therapy (Corre et al., 2020).

Epigenetic modification has been globally investigated in OS (Nebbioso et al., 2018). Almost all types of epigenetics, ranging from DNA methylation to histone modification, have been suggested to involve in the development and progression of OS (Cui et al., 2011). For instance, DNA methylation could downregulate miR-449c expression and eventually contributed to the tumorigenesis of OS (Li Q. et al., 2017). Meanwhile, histone methyltransferase has been discovered to regulate the chemosensitivity in OS (Jiang et al., 2018; He et al., 2019). Additionally, potential roles of multiple ncRNAs also have been validated in OS, such as the circRNA (Tu et al., 2020) and lncRNA (Ren et al., 2020; Xu et al., 2020). Li X. et al. (2017) reported that HOX transcript antisense intergenic RNA (HOTAIR) could enhance the development of OS via DNA methylation of CDKN2A gene. Notably, there have been some indications that m^6^A modification exerts pivotal functions in OS.

An integrative study performed by Wang J. et al. (2019) analyzing the transcriptome-wide m^6^A methylome enriched by chemotherapy in OS stem cells (OSCs) has revealed that several m^6^A-related enzymes (METTL3, METTL14, FTO, ALKBH5) were altered in OS cells compared with noncancerous counterparts. The aberrantly expressed genes were associated with pluripotency regulation of the OSCs (Wang J. et al., 2019). Further, it has been shown that lncRNA plasmacytoma variant translocation 1 (PVT1) transcript was upregulated because of m^6^A methylation decrease mediated by ALKBH5, which reduced the binding of reader protein YTHDF2 in PVT1, subsequently lessening the degradation of PVT1 and promoting tumorigenesis of OS (Chen et al., 2020). METTL3 was found localizing in both cytoplasm and nucleus of OS cells, which is also on the map of OS modulation. Downregulation of METTL3 was reported to suppress the expression of ATPase family AAA domain containing 2 (ATAD2), in conjunction with inhibition of OS cell growth and metastasis (Zhou et al., 2020). In addition, knockdown of METTL3 has been demonstrated to be associated with decreased m^6^A methylation of GTP-binding protein (DRG) 1. Concomitant with the decreased m^6^A methylation, the stability and expression level of DRG1 were reduced (Ling et al., 2020), resulting in suppression of the OS development, migration, and colony formation. Meanwhile, silence of METTL3 decreased the m^6^A methylation and expression of lymphoid enhancer binding factor 1 (LEF1), followed by the advent of Wnt/β-catenin signaling pathway deactivation. Consequently, aberration of Wnt/β-catenin pathway contributed to the development of OS (Miao et al., 2019).

Collectively, based on the current evidences, the m^6^A writers, mainly the METTL3, are found extensively involved in the tumorigenesis, progression, and migration of OS via methylating their target genes. However, the current recognition is still restricted because of the limited number and depth of studies.

### M^6^A in OA

OA is the most prevalent chronic joint disease that mainly occurs in aging and obesity population. The prevalence of OA is still in continuous growth, and it is estimated that the proportion of OA in population 45 years or older will increase from 26.6 to 29.5% by 2032 (Hunter and Bierma-Zeinstra, 2019). Symptoms of OA typically include stiffness, pain, and movement restriction with a high risk of disability, which can bring substantial socioeconomic burden (Hunter et al., 2014). The pathogenesis of OA principally involves in degradation of cartilage matrix, which consists of collagen type II, minor collagen types IX and XI, and gel-like negatively charged proteoglycans (Kannu et al., 2009). Likewise, many inflammation-related molecules have been suggested to engage in this process such as the growth factors [transforming growth factor β, fibroblast growth factor 2 (FGF-2), and FGF-18], Wnt, β-catenin, HIF-2α, etc. (Xia et al., 2014). Targeting the inflammation pathways is regarded as a promising way for OA therapy. Particularly, epigenetic regulation has been reported to be in connection with the inflammatory factors and response (Shen et al., 2017). As an indispensable way of epigenetic regulation, m^6^A has also been partially studied in OA.

It was expounded that METTL3 regulated OA process via enhancing inflammatory response and extracellular matrix (ECM) synthesis (Liu Q. et al., 2019). Mechanistically, silencing METTL3 inhibited the inflammatory cytokines level and nuclear factor κB signaling in OA cells, thereby deactivating the progression of OA. Meanwhile, suppression of METTL3 boosted the degradation of chondrocytes ECM through downregulating the matrix metalloprotease-13 and collagenase type X, consequently promoting the development of OA (Liu M. et al., 2019). In addition, the association between OA and FTO has also been explored in some genome-wide association studies in which they clarified that FTO-mediated overweight increased the susceptibility of OA (Zeggini et al., 2012; Panoutsopoulou et al., 2014). However, another study performed by Dai et al. (2018) showed that the FTO polymorphism (rs8044769) was not linked to OA in the Chinese Han population, and their association may be mediated by other genes. Therefore, the correlation between FTO and OA remains elusive and requires further exploration.

### M^6^A in RA

With a symptom of pain, swelling, and stiffness, RA is a common chronic inflammatory disease that primarily attacks the synovial joint (Littlejohn and Monrad, 2018). RA can bring substantial burden to both the individuals and socioeconomics because of its high morbidity and mortality (Hu et al., 2018). Besides, RA is in close linkage with the occurrence of cardiovascular diseases (Blum and Adawi, 2019). Autoimmune-mediated inflammation is a well-known cause of RA (Derksen et al., 2017), in which both the genetic regulation (Scott et al., 2010) and epigenetic regulation (Doody et al., 2017) are proposed to play indispensable roles.

The relationship between m^6^A modification and RA has been partially unraveled in several studies. A large-scale genome-wide association study identifying the m^6^A-associated SNPs (m^6^A-SNPs) that affected the progression of RA has been conducted. Thirty-seven RA-related m^6^A-SNPs were discovered, and 27 of them were verified to affect expression of 24 local genes in different RA cells or tissues, which indicated the potential roles of m^6^A-SNPs in RA (Mo et al., 2018b). Moreover, METTL3 was validated to significantly suppress the inflammatory response of macrophages in RA. Specifically, METTL3 inhibited the generation of IL-6 and TNF-α in macrophages via restraining the phosphorylation of NF-κB. Therefore, METTL3 may serve as a potential biomarker for diagnosis of RA (Wang J. et al., 2019). However, because of the limitation of current studies, we can only hypothesize that m^6^A can decelerate the progression of RA via regulating the inflammatory response of immune cells; studies are still needed to provide more evidences.

### M^6^A in OP

Characterizing by depletion of bone mass and impairment of bone structure, OP is mostly a condition of postmenopausal women and aging, which may lead to disastrous fracture in some cases (Armas and Recker, 2012; Boyanov et al., 2014).

Several researches have enhanced our recognition of the roles of m^6^A modification in OP. A genome-wide identification study was indicative of the potential roles of m^6^A-SNPs in bone mineral density. The results revealed that 138, 125, and 993 m^6^A-SNPs were in linkage to femoral neck density disorders, lumbar spine density disorders, and heel density disorders, respectively (Mo et al., 2018a). Currently, it becomes obvious that factors associated with regulation of BMSCs differentiation are closely associated with OP. The imbalance between BMSC-derived osteoblasts and adipocytes was deemed underlying progression of OP (Chen Q. et al., 2016). It has been demonstrated that METTL3-mediated m^6^A methylation affected the function of BMSCs through several pathways. In the first place, knockdown of m^6^A methyltransferase METTL3 in mice induced pathological features related to the occurrence of OP via decreasing parathyroid hormone (PTH)/parathyroid hormone receptor-1 (Pth1r) signaling axis, interfering the PTH-induced osteogenic response of BMSCs (Wu et al., 2018). Moreover, downregulation of METTL3 in BMSCs inhibited the methylation of runt-related transcription factor 2 (RUNX2) and precursor (pre-) miR320 (Yan et al., 2020). RUNX2 is an essential regulator of osteoblast progenitor proliferation and osteogenic differentiation, which can enhance the bone mineralization, and multiple factors were reported to participate in the osteogenic process via targeting RUNX2 (Hou et al., 2019; Komori, 2019).

Conversely, the FTO, represented as the RNA demethylase, was reported to promote the shift of osteoporotic BMSC fate to adipocyte and impede the bone formation through a growth differentiation factor 11 (GDF11)-FTO-peroxisome proliferator-activated receptor γ (Pparg) axis, whereby high FTO expression indicated high risk of OP (Shen et al., 2018). Meanwhile, the expression of FTO could be repressed by overexpression of miR-149-3p, followed by a higher potential of BMSCs to differentiate into adipocytes (Li et al., 2019b). In addition, of SNPs in multitude utilizing the association analyses, it was shown that a FTO such as rs1421085, rs1558902 and rs1121980 were associated with bone mineral density and risk of fracture (Guo et al., 2011; Tran et al., 2014). Interestingly, although FTO inhibited the BMSCs from differentiating into osteoblasts, it could exert a protective role in differentiated osteoblasts. Osteoblasts with FTO suppression were prone to develop cell death, which was illustrated to be caused by the interruption of DNA repair pathway. Specifically, FTO was able to stabilize the endoplasmic reticulum stress pathway components, such as Hsp70, protecting osteoblasts from genotoxic damage (Zhang Q. et al., 2019).

Taken together, m^6^A modification is involved in the occurrence and development of OP via (1) METTL3-mediated differentiation of BMSCs to osteocyte, (2) FTO-mediated differentiation of BMSCs to adipocyte, and (3) FTO-mediated protection of osteoblasts from genotoxic damage. Herein, we assume that m^6^A may play a dual role in OP, by which it can either promote or decelerate the progression of OP via different modifications.

## Clinical Utilizations of M^6^A in Musculoskeletal Disorders

RNA target therapy has become a hotspot and shown convincing prospects in treatment of many diseases with high specificity and efficacy (Crooke et al., 2018). The broad involvement of m^6^A in musculoskeletal disorders, as outlined previously, has driven extensive research efforts at m^6^A-based therapy. The functions of the m^6^A regulatory proteins including FTO, METTL3, ALKBH5, etc. have been determined in OS, OA, RA, and OP as mentioned previously. As a result, the possibility is then opened for developing inhibitors or promoters of them to control the diseases.

Some natural products have been discovered showing significant activity in FTO inhibition. The natural product rhein is the first identified small-molecule inhibitor of human FTO demethylase, which competitively binds to the FTO active site and inhibit the demethylation *in vitro* (Chen et al., 2012). Additionally, by using the structure-based hierarchical virtual screening, researchers have found that entacapone directly bound to FTO and subsided its activity (Peng et al., 2019). And the natural compound radicicol also has been recognized as a potent FTO inhibitor, which suppressed the FTO demethylation activity in a dose-dependent manner (Wang et al., 2018). Besides, the meclofenamic acid (MA), a nonsteroidal anti-inflammatory drug, was identified as a specific inhibitor of FTO. Mechanically, MA competed with FTO for the binding sites within the m^6^A modified RNA, reducing activity of FTO-mediated demethylation (Huang et al., 2015). Further, FTO may interfere the reaction of human body to other drugs. For instance, it has been elucidated that the rs7195994 variant at the FTO gene locus hereditarily impacted the TNF inhibitor response in RA patients, and the customized treatment based on the FTO genetic stratification of patients could improve the efficacy (Massey et al., 2018).

Similarly, because METTL3 up-regulation in OS, OA, and RA contributes to the progression of diseases, METTL3 may also be targeted for treatment. Although METTL3 inhibitor is not available so far, it provides us with a novel direction. The fact that several drug screening technologies for RNA-modifying enzymes such as the self-assembled monolayer desorption/ionization have been developed illustrates a promising future for METTL3-based drugs (Buker et al., 2020). Recently, the first series of small molecule inhibitors of METTL3 have been identified via high-throughput docking into the SAM binding site and protein X-ray crystallography, in which seven compounds belonging to N-substituted amide of ribofuranuronic acid analogs of adenosine or adenosine mimics with a six-member ring were uncovered to be the effective METTL3 inhibitors (Bedi et al., 2020).

In summary, m^6^A-modifying proteins can serve as potential targets for drugs with which the FTO and METTL3 inhibitor may have great prospect in the treatment of musculoskeletal disorders.

## Discussion

Epigenetics has been widely illuminated in multiple diseases over the past decades, and researchers are consistently seeking for new remedy from this field (Prachayasittikul et al., 2017). Currently, a diverse set of RNA modifications has been identified and annotated. Of note, m^6^A is the most abundant among them (Linder et al., 2015). Even though the recent advances have highlighted the crucial role of m^6^A in a multitude of diseases, only a small percentage of them focus on the musculoskeletal disorders. In this review, we have discussed the molecular mechanisms, detection technologies, regulatory functions, and clinical implications of m^6^A in musculoskeletal diseases. As we summarized previously, m^6^A modification is of great potential in disease prediction and drug development, yet the current studies are insufficient.

The first issue is how to choose and optimize the laboratory used technology for m^6^A detection in clinical practice. Although a diverse set of methods have been continually developed in experiment to detect the m^6^A modification or analyze the m^6^A residue locations within RNA site over the past decades, the use in clinic has not been investigated yet. Here, we envision that we can associate the disease progression with the m^6^A presence patterns that may be presented as the presence of m^6^A, the proportion of m^6^A, or the specific site of m^6^A. With the appropriate detection method, doctors will be able to figure out the situation of musculoskeletal diseases through the m^6^A examination results.

Furthermore, it has remained a significant challenge to dissect the mechanism of m^6^A in musculoskeletal disorders because limited studies have been performed toward it, especially for OA and RA. Meanwhile, the current studies of m^6^A in musculoskeletal disorders focus only on the “writers” and “erasers,” whereas clues toward the “readers” are scarce. As a matter of fact, roles of the “readers” have been investigated in other diseases. For instance, high YTHDF1 expression was a significant predictor of malignant tumor behaviors and poor prognosis in colorectal cancer (Nishizawa et al., 2018). IGF2BPs was demonstrated to participate in suppression of glycolysis and stemness properties of breast cancer cells via a FGF13-AS1/IGF2BPs/Myc feedback loop (Ma et al., 2019). Thus, the “readers” may also engage in the pathophysiological processes of musculoskeletal disorders.

Besides, m^6^A-based drugs remain poorly understood. Only a limited number of FTO and METTL3 inhibitors have been identified, yet their efficacy and safety are inconclusive. Notably, there are currently no m^6^A-based drugs developed for musculoskeletal disorders. Therefore, to address these limitations, we still have a long way to go.

## Conclusion

As the most abundant RNA modification in eukaryotic cells, it is beyond doubt that m^6^A is a central node of the regulatory network of diseases. The illustration of m^6^A function has revealed its great importance in both the biological and pathological processes of bone and skeletal muscle. M^6^A modification has been partially studied in musculoskeletal disorders, including OS, OA, RA, OP, etc. Regarding its pivotal role in regulating the progression and development of diseases, m^6^A modification is of great potential to serve as the diagnostic biomarker or therapeutic target in musculoskeletal diseases, although more evidences are still warranted for validation in the future. Furthermore, a growing number of technologies have been developed for the m^6^A detection, and it has become evident that m^6^A is detectable and usable in disease prediction. Even with all of the effort over the recent years to figure out the detection methods for m^6^A, it should be noted that there is no clear indication for selecting the most suitable detection method for clinical application. Simplified m^6^A detection methods with high specificity/sensitivity and low costing, such as dot blot, immuno-Northern blot, and electrochemical immunosensor method, may be the promising methods to be utilized.

## Author Contributions

WZ and CT conceived and designed the work. WZ, LH, and ZLiu contributed to material preparation and performed data collection and analysis. WZ wrote the first draft of the manuscript. XR, LQ, LW, and WW wrote sections of the manuscript. All authors commented on previous versions of the manuscript. CT revised the manuscript. ZLi contributed to manuscript drafting, critical revision, and final approval of the version to be published. All authors read and approved the final manuscript.

## Disclaimer

Figures of this review were created with the aid of Servier Medical Art (https://smart.servier.com/), reproduced under Creative Commons License attribution 3.0 Unported License.

## Conflict of Interest

The authors declare that the research was conducted in the absence of any commercial or financial relationships that could be construed as a potential conflict of interest.
